# Red Orange and Bitter Orange IntegroPectin: Structure and Main Functional Compounds

**DOI:** 10.3390/molecules27103243

**Published:** 2022-05-19

**Authors:** Antonino Scurria, Marzia Sciortino, Ana Rosa Garcia, Mario Pagliaro, Giuseppe Avellone, Alexandra Fidalgo, Lorenzo Albanese, Francesco Meneguzzo, Rosaria Ciriminna, Laura M. Ilharco

**Affiliations:** 1 Istituto per lo Studio dei Materiali Nanostrutturati, CNR, Via U. La Malfa 153, 90146 Palermo, Italy; antonino.scurria@ismn.cnr.it; 2 Dipartimento DICEAM, Università degli Studi “Mediterranea” di Reggio Calabria, Via Graziella, Loc. Feo di Vito, 89122 Reggio Calabria, Italy; 3 Dipartimento di Scienze e Tecnologie Biologiche Chimiche e Farmaceutiche, Università di Palermo, Via Archirafi 32, 90123 Palermo, Italy; marziasciortino@gmail.com (M.S.); beppe.avellone@unipa.it (G.A.); 4 Institute of Bioscience and Biotechnology, Instituto Superior Técnico, Universidade de Lisboa, Avenida Rovisco Pais 1, 1049-001 Lisboa, Portugal; argarcia@tecnico.ulisboa.pt (A.R.G.); alexandra.m.abrantes.fidalgo@gmail.com (A.F.); 5 Istituto per la Bioeconomia, CNR, Via Madonna del Piano 10, 50019 Sesto Fiorentino, Italy; lorenzo.albanese@cnr.it (L.A.); francesco.meneguzzo@cnr.it (F.M.)

**Keywords:** IntegroPectin, citrus, hesperidin, naringin, green extraction, hydrodynamic cavitation

## Abstract

DRIFT, HPLC-MS, and SPME-GC/MS analyses were used to unveil the structure and the main functional compounds of red (blood) orange (*Citrus sinensis*) and bitter orange (*Citrus aurantium*). The IntegroPectin samples show evidence that these new citrus pectins are comprised of pectin rich in RG-I hairy regions functionalized with citrus biophenols, chiefly flavonoids and volatile molecules, mostly terpenes. Remarkably, IntegroPectin from the peel of fresh bitter oranges is the first high methoxyl citrus pectin extracted via hydrodynamic cavitation, whereas the red orange IntegroPectin is a low methoxyl pectin. *C. aurantium* IntegroPectin has a uniquely high concentration of adsorbed flavonoids, especially the flavanone glycosides hesperidin, naringin, and eriocitrin.

## 1. Introduction

Chiefly derived from citrus peel and apple pomace, pectin is the most valued food hydrocolloid [1]. New developments in pectin science and chemical technology (new green chemistry extraction and new green engineering isolation technologies) have opened the route to new and unexpected applications of this uniquely complex heteropolysaccharide, including the usage of pectin extraction byproducts, particularly pectin oligosaccharides and polyphenols, thereby broadening the applicability of pectin [2].

Among the aforementioned new green extraction technologies, hydrodynamic cavitation (HC) of citrus fruit biowaste obtained after citrus juice industrial extraction has lately emerged as one of the most promising natural product green extraction techniques [3]. Carried out directly in water at a semi-industrial scale (42 kg of waste orange peel in 120 L water), the process affords a low methoxyl (LM) pectin with 17.05% degree of esterification (DE, percent of methyl-esterified carboxyl groups) uniquely rich in adsorbed hesperidin, naringin (and hydroxycinnamic acid derivatives) alongside limonene, myrcene, and α-pinene [4]. 

The process has been subsequently extended to other citrus processing biowastes, including those of lemon [5] and grapefruit [6]. Invariably, the citrus pectin isolated after the HC-based extraction via freeze-drying contained large amounts of citrus flavonoids and terpenes, with a uniquely low DE. Accordingly, we dubbed these whole citrus pectins “IntegroPectin” to differentiate them from conventional citrus pectin industrially extracted via conventional acid hydrolysis of citrus or apple juice processing waste.

The latter is a high-methoxyl (DE > 50%), highly degraded form of natural pectin, having lost during hydrolysis most “hairy” rhamngalacturonan (RG-I and RG-2) regions during the acid hydrolytic extraction, leaving most of the “smooth” homogalacturonan (HG) regions with a few neutral sugar units bound to the main galacturonic acid chain [7]. Beyond use as a hydrocolloid and texturizing agent in the food and beverage industries, commercial pectin finds applications in, for example, the biomedical industry where it is chiefly used as a biocompatible glue [8].

Herein, we now report the outcomes of the first structural and chemical investigations of IntegroPectin biopolymers obtained from bitter orange and blood orange biowaste (referred as IP-bitter and IP-blood, respectively) by the aforementioned HC process carried out in water only (i.e., without the addition of chemical reactants or organic solvents). The structure of the pectin samples was characterized by diffuse reflectance infrared Fourier transform (DRIFT) spectroscopy. The high-performance liquid chromatography–mass spectrometry (HPLC–MS) technique was used to detect and quantify biophenols in hydroalcoholic extracts. Volatile compounds in both *Citrus* pectins were identified by solid-phase microextraction (SPME) gas chromatography coupled to mass spectrometry (SPME-GC/MS).

Characterized by numerous health-beneficial bioactive compounds such as flavonoids (mainly hesperidin in the juice and peel), limonoids (limonoid glucosides abundant in juice and pulp and limonoid aglycones in the seeds) and anthocyanins (malvidin-3-*O*-glucoside), blood orange is a highly valued sweet orange (*Citrus sinensis*) variety [9]. Not used as an edible fruit due to its bitter and sour taste, bitter orange (*Citrus aurantium*), in its turn, is particularly rich in phenolic acids (mainly ferulic acid and *p*-coumaric acid) contained both in the pulp and in the peel [10], alongside naringin, neohesperidin, and rutin chiefly contained in the peel [11]. Finally, concentrated in the peel of bitter oranges are high levels of a protoalkaloid and *p*-synephrine, with sympathomimetic activity and high lipolytic action in human adipose tissue, thanks to which bitter orange standardized extracts are used as a safe (at the commonly used doses) weight loss/weight management dietary supplement [12].

## 2. Materials and Methods

### 2.1. Biophenol-Rich Extract Preparation

Both IntegroPectin materials were obtained using the HC-based extractor previously described in detail [4]. For red (blood) orange IntegroPectin, a 40 kg sample of industrial waste orange peel kindly donated by OPAC Campisi Società Cooperativa Agricola (Siracusa, Italy) was processed. For bitter orange IntegroPectin, fresh oranges owned by one of the authors (A.S.) were collected and peeled by hand, and the resulting orange peel waste was processed via HC under the same conditions previously used to process orange peel waste [4]. Following cavitation, aliquots of the resulting aqueous mixture were filtered to remove the cellulosic insoluble fraction after which the resulting clear solution was freeze-dried using a Labconco (Kansas City, MO, USA) FreeZone 4.5 lyophilizer. The resulting IntegroPectin samples were extracted with aqueous ethanol. In detail, for every material a 600 mg aliquot was extracted in a 100 mL flask with a 50 mL aliquot of EtOH/H_2_O (4:1, *v*/*v*) solution. The extraction was carried out under sonication (150 W) for 15 min using a Transsonic 460 H ultrasonic bath (Elma Hans Schmidbauer, Singen, Germany) operating at 35 kHz. During extraction, the temperature was monitored with a thermometer placed inside the ultrasonic bath. Figure S1 in the Supplementary Materials shows evidence that both IntegroPectin samples remain colored even after the extraction.

After sonication, the content of each flask was transferred to a centrifuge tube to undergo centrifugation at 10,000 rpm for 10 min in an Allegra (Beckman Coulter, CA, USA) X-22 R centrifuge. A small sample of the supernatant of both samples was thus added to glass vials for analysis. In order to check whether, and to what extent, the room temperature extraction leaves unextracted biophenols, for *C. aurantium* IntegroPectin a subsequent extraction of the pectin sample extracted at room temperature was conducted with the same aqueous ethanol mixture, but this time at 90 °C.

### 2.2. DRIFT Analysis

The DRIFT spectroscopy investigation was carried out using a Bruker (Billerica, MA, USA) Vertex 70 FTIR spectrometer equipped with a wide band MCT detector and a Specac selector, in the range 4000 to 500 cm^−1^, at 4 cm^−1^ resolution. The spectra were the result of ratioing 200 co-added single beam scans for each sample (ground pectin powder diluted in ground FTIR-grade KBr, in the appropriate proportion to assure the validity of the Kubelka–Munk assumptions) [13] against the same number of scans for the background (ground KBr). The spectra were transformed to Kubelka–Munk units using the OPUS software (Bruker Optics, Ettlingen, Germany) and further processed using the Origin software (OriginLab Corporation, Northampton, MA, USA).

### 2.3. HPLC–MS Analysis

The HPLC–MS analyses were conducted using an Alliance e2695 (Waters, Milford, MA, USA) HPLC system equipped with autosampler, degasser, and a column heater coupled with a Q-Tof Premier (Waters, Milford, MA, USA) quadrupole time-of-flight mass spectrometer. The compounds were separated by a Thermo Scientific (Thermo Fisher Scientific, Waltham, MA, USA) Hypersil Gold HPLC C18 column (50 mm, 2.1 mm I.D., particle size 1.9 µm), kept at 20 °C, that injects a 5 µL volume sample. All samples were injected in duplicate using a thermostat autosampler kept at 4 °C. The HPLC analyses were carried out using a mixture of 0.1 wt% aqueous formic acid and 0.1 wt% formic acid methanolic solution at 0.25 mL/min flow rate. Elution started with 95% aqueous formic acid and 5% methanol formic acid that was maintained isocratic for one minute. In the subsequent 14 min, the solvent becomes 100% MeOH, remaining isocratic for the subsequent 5 min (from min 15 to min 20). After 30 s, the eluting solvent mixture is reverted to 95% aqueous formic acid and 5% methanolic formic acid, and kept as such for another 30 s. The whole run lasted 21 min. Every sample was injected twice. The concentration values reported in the following are the average of the two values measured.

Quantification of naringenin, naringin, hesperidin, eriocitrin, and gallic acid was carried out using commercial samples of the phenolic compounds obtained from Sigma Aldrich (Gallarate, Italy) as standards. For the remaining compounds, we used the calibration curve of naringin for the detection of flavonoid glycosides, the calibration curve of naringenin for the detection of flavonoids, and the calibration curve of gallic acid for the assessment of other biophenols.

The MS experiments were performed on Q-Tof Premier using dynamic range enhancement (DRE) as the acquisition mode that avoids MCP saturation, maintaining a fairly good sensitivity. This allows to correctly quantify very abundant as well as trace level compounds, providing results suitable for statistical analysis. Atmospheric pressure electrospray ionization (ESI) in negative mode was used under the following conditions: capillary, 2.0 kV; extraction cone, 2.0 V; ion guide, 2.0 V; source temperature 80 °C; cone gas, N_2_, flow 35 L h^−1^; and desolvation gas, N_2_, flow 300 L h^−1^. Figures S2–S6 in the Supplementary Materials display the LC chromatograms for both IntegroPectin hydroalcoholic extracts along with the mass spectra of selected biophenols identified in said extracts.

### 2.4. SPME-GC/MS Analysis

The SPME-GC/MS experiments were carried out by performing the GC–MS analysis of the volatile compounds released in the headspace of a closed vial upon SPME using a thin polymer coating fixed to the solid surface of a DVB/CAR/PDMS fiber. This enabled the direct identification of the volatile compounds by comparison with the mass spectra of authentic standards. A Thermo Fisher Scientific (Waltham, MA, USA) TSQ 800 triple quadrupole mass spectrometer and a Thermo Fisher Scientific Trace 1310 gas chromatograph analyzer were used for the GC–MS analyses. The analytical conditions used appear in Table 1. The injections took place in splitless mode at 260 °C. The mass analysis was carried out under conditions of electronic ionization (EI) with an ionization energy of 70 eV.

The IntegroPectin samples were left in closed vials at room temperature to equilibrate for 24 h before sampling. The extraction of volatile components accumulated in the headspace took place by injecting the coated fiber in the headspace and leaving the fiber to adsorb the volatiles for 2 min in the case of red orange pectin, and 20 s in the case of bitter orange pectin. After that, the fiber was withdrawn from the vial and inserted into the hot GC injector. In order to avoid carry over effects or artifact formation, blank runs were carried out every three analyses. Prior to use, and between consecutive analyses, the fiber was conditioned in a GC injector at 260 °C for 3 h to remove contaminants. Nonetheless, siloxane contaminants most likely derived from the injector led to the appearance of interference siloxane peaks in the chromatogram that were readily identified.

The fiber used for sampling both bitter and red orange pectin used a divinylbenzene/carboxen/polydimethylsiloxane (DVB/CAR/PDMS, 50 µm the DVB layer and 30 µm the CAR/PDMS layer) coating suitable for analyte group flavors (volatiles and semivolatiles). In order to assess the best extraction time for this coating, several preliminary tests at increasing extraction times (20 s, 1 min, 2 min, and 5 min) were investigated (data not shown). The 2 min microextraction time was identified as optimal to reproduce the extraction procedure for red orange pectin and the 20 s time was chosen for bitter orange pectin. Attribution of compounds corresponding to the chromatographic peaks was performed by comparing the corresponding mass spectra with those of the NIST 11.0 and Wiley 7.0 mass spectrum libraries, verifying the fragmentation through a careful study of the EI spectra. Furthermore, the results were confirmed using the following standard compounds purchased by Sigma Aldrich (Gallarate, Italy): α-pinene, 1-pentanol, *trans*-linalool oxide, 2,3-butanediol, α-linalool, 1,3-butanediol, α-terpineol, *d*-limonene, 1,3-bis(1,1-dimethylethyl)-benzene, 1-hexanol, phenylethyl alcohol, and 3-hexen-1-ol. 

## 3. Results and Discussion

### 3.1. DRIFT Structural Analysis

**Figure 1:**
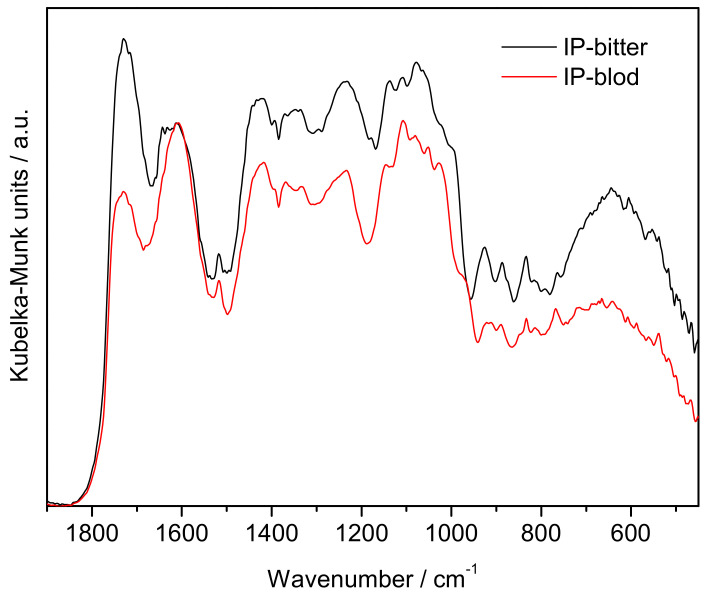
DRIFT spectra of the pectin samples in the 500–2000 cm^−1^ region, normalized to the ν_as_COO^−^ band carboxylate groups, at ~1610 cm^−1^.

The DRIFT spectra in Figure 1 (500–2000 cm^−1^ region) show the typical features of pectin, with differences in the relative intensities and frequencies of some fingerprint bands.

The strong bands in the 1550–1800 cm^−1^ region, with maxima at 1610, 1643 (IP-bitter) and 1730 cm^-1^, are assigned to the stretching modes of carboxylate groups (ν_as_COO^−^), carbonyl groups from non-esterified hydrogenated acidic carbonyl groups (νC=O_acid_) and esterified galacturonic acid (νC=O_ester_), respectively [14]. In this region, the relative intensities of the ester-related components are much higher for IP-bitter pectin, suggesting a higher degree of esterification.

The 1200–1550 cm^−1^ region is dominated by CH_x_ and C-O-H deformation modes, partially overlapped with ester-related stretching modes [15,16]. These include: the δ_ip_C-O-H (from alcohol hydroxyl groups in the pyranose rings of the pectin chain) at ~1236 cm^−1^, the νC-O-C_ester_ at ~1330 cm^−1^, the ν_s_COO^−^ at ~1420 cm^−1^, and the δ_s_CH_3_ and δ_as_CH_3_ (from ester methyl groups in the galacturonic rings and rhamnose rings of the pectin backbone) at 1336 and 1520 cm^−1^.

The 950–1200 cm^−1^ region contains a set of very intense bands typical of pectin, partially overlapped, assigned to skeletal stretching modes (νC-C and νC-O-C) of the pyranose rings and of the glycosidic bonds, and to a combination of the νC-OH and νC-C modes from the pyranose rings [17,18]. The 500–950 cm^−1^ region contains the bands related to the external deformation vibrations of methyl, methylene, and methine groups (ρCH_x_ and δC-H) [14].

The degree of esterification of each pectin (percent of esterified carboxyl groups) was obtained by spectral analysis of the 1550–1800 cm^−1^ region, as the ratio of ester carboxyl to total carboxyl peak areas (Equation (1)) [15,19]:

DE = Σ*A*_νC=Oester_/(Σ*A*_νC=Oester_ + *A*_νC=Oacid_ + *A*_νasCOO_^−^) (1)

The amount of galacturonic acid-rich (HG) regions of pectin are not calculated, but they are proportional to the ratio described by Equation (2) [15]:

HG α (Σ*A*_νC=O_ + *A*_νasCOO_^−^)/(Σ*A*_νC-O-C_ + Σ*A*_νC=O_ + *A*_νasCOO_^−^) (2)

The νC=O and νasCOO^−^ band areas of the samples were estimated by decomposing the 850–1800 cm^−1^ region into a sum of Gaussian components, using a nonlinear least-squares fitting [15]. The components’ centers and integrated areas are summarized in Table 2 for the two samples, as well as the values obtained for DE and to quantities proportional to HG regions.

Although both pectins were extracted by the same procedure (though bitter oranges were not industrially squeezed to extract the juice), the IntegroPectin from bitter orange is an HM pectin with DE = 59%, whereas blood orange IntegroPectin is a LM pectin with DE of 32%. This outcome has known consequences in terms of applications and demand, as LM pectins tend to form gels electrostatically, in the absence of sugar and in a broad pH range (2–7), in the presence of a small amount of divalent cations such as Ca^2+^ [20]. The outcome is another proof of the dependence of DE on the extraction method, but also on the fruit source and its processing [21].

The DE of blood orange waste IntegroPectin, in its turn, is close to that of pectin extracted from blood orange waste via microwave-assisted hydrodiffusion in water (DE = 25%) under acid-free conditions [21].

For comparison, pectin extracted from blood orange peel at pH 3 and at 100 °C has a high degree of methylation (>60%) and 81% galacturonic acid content [22]. This shows that the HC-based extraction acts similarly to extraction in a single-mode microwave cavity system which promotes rapid pectin de-esterification [23]. Indeed, also in the case of bitter orange, when the extraction is carried out at pH 1.5 under microwave irradiation, the bitter orange pectin obtained is an LM pectin (DE of 1.5%) [24].

The HC-based extraction, on the other hand, prevents the hydrolytic loss of the RG-I regions, particularly in the case of pectin derived from blood orange biowaste. Both pectins obtained, indeed, are rich in smooth HG regions, with bitter orange IntegroPectin having a substantially higher share of HG regions (71% vs. 63%). This points to higher quality of blood orange IntegroPectin not only because the latter is a valued LM pectin, but also because pectin molecules richer in hairy branched chains have a better and longer emulsifying activity, forming a thicker interfacial layer on the oil–water interface [25]. The structure, for example, provides stronger steric hindrance, promoting the long-term stability of the emulsion [26].

### 3.2. Biophenols

Table 3 lists the flavonoid and other phenolic compounds found using the highly sensitive HPLC–MS technique employed in red orange IntegroPectin, bitter orange IntegroPectin, and bitter orange IntegroPectin re-extracted at 90 °C.

Results in Table 3 clearly show the abundance of phenolic compounds in both orange IntegroPectin samples, with a uniquely high concentration found in bitter orange IntegroPectin, amounting to 119.60 mg/g, namely nearly seven times higher than the amount found in red orange IntegroPectin. Furthermore, it is enough to treat the bitter orange IntegroPectin extracted with aqueous EtOH with another aliquot of the same solvent at higher temperature (90 °C), to extract another significant amount (19.22 g/kg) of biophenols.

Hesperidin (hesperetin 7-rutinoside), naringin (4′,5,7-trihydroxyflavonone-7-rhamnoglucoside), and eriocitrin (eriodictyol 7-rutinoside) are, by far, the most abundant compounds found in the bitter orange pectin. Hesperidin and naringin are the two main flavonoids found in the red orange pectin samples. Remarkably, and pointing to the well-known poor solubility of hesperidin and naringin in protic solvents [27], a significant amount of the latter flavanone glycosides can be extracted at 90 °C from the bitter orange pectin sample already extracted at room temperature.

Diosmin (diosmetin 7-O-rutinoside) was only detected (0.285 mg/g) in the bitter orange pectic extract, whereas rutin (quercetin-3-O-rutinoside) was found (0.131 mg/g) only in the red orange pectic sample.

From a compositional viewpoint, these results are in agreement with previous findings for which bitter oranges possess a unique flavanone profile dominated by naringin, neoeriocitrin, and neohesperidin, with a total flavanone aglycones amount of about 48 mg/100 g fruit, significantly higher than that of sweet oranges (about 17 mg total flavanone aglycones/100 g) [28]. However, the amount of flavanone found at the surface of both citrus IntegroPectin samples is three orders of magnitude higher in the case of the IntegroPectin samples than in the fruit peel. This, once again, is due to the adsorption and concentration process taking place during the HC-based extraction process followed by freeze-drying of the aqueous extract rich in flavonoids released into the aqueous phase most likely with pectin acting as emulsifying agent. For comparison, the overall amount of biophenols found in grapefruit IntegroPectin, a powerful and broad-scope antibacterial agent [6], was about 75 mg/g [29]. The high amount of naringin (47.16 mg/g), about half of that found in grapefruit IntegroPectin (73.66 mg/g), imparts the bitter taste to *C. aurantium* due to the sugar neohesperidose, in contrast to rutinose of hesperidin that causes these flavanones to have a neutral taste [30].

Remarkably, the amount of eriocitrin found in *C. aurantium* IntegroPectin (14.76 mg/g) is nearly five times higher than in lemon IntegroPectin (3.35 mg/g) [29]. Supported by clinical studies in pre-diabetic patients showing benefits in glycemic control, reduced systemic inflammation, and oxidative stress [31], new nutraceutical products using lemon-derived eriocitrin as the active ingredient for reducing blood glucose levels have been lately commercialized [32].

The uniquely high amount of hesperidin (56.81 mg/g) in bitter orange IntegroPectin vs. 15.09 mg/g in red *C. sinensis* IntegroPectin is particularly promising in view of biomedical applications. Being the most widely investigated citrus flavonoid in biomedical research, hesperidin shows a broad spectrum bioactivity spanning from the prevention of cancer and cardiovascular diseases [33] to neurodegenerative diseases such as Parkinson’s, Alzheimer’s, Huntington’s, and multiple sclerosis [34].

### 3.3. Volatile Compounds

The results of the GC analysis are summarized in Table 4, displaying the main analytes molecular weight (rounded to the nearest integer) and fragment ions in both orange IntegroPectin samples.

Table 5 lists the fourteen volatile compounds identified in the case of bitter orange pectin alongside their relative abundance. Seven compounds are present only in trace amounts. Table 6 displays the eight volatile compounds found in red orange IntegroPectin.

Highly bioactive monoterpene α-terpineol is by far (82.6%) the most abundant volatile molecule found in *C. aurantium* IntegroPectin [35]. The presence of aliphatic alcohols 1,3-butanediol (7.26%) and 2,3-butanediol (5.16%) suggests likely microbial fermentation of sugars abundant in the citrus peel (note that, in the case of *C. aurantium*, the fruits were not squeezed and the peel was removed by hand) [36]. Anti-inflammatory, antidiabetic, antioxidant, and anticarcinogenic limonene [37], in its turn, is by far the dominant volatile molecule of red orange IntegroPectin, with a percentage approaching 91%. For comparison, α-terpineol and terpinen-4-ol are the dominant terpenes in lemon IntegroPectin [5], whereas α-terpineol and linalool are the dominant terpenes in grapefruit IntegroPectin [5].

## 4. Conclusions

The DRIFT, HPLC–MS and SPME-GC/MS analyses of red (“blood”) and bitter orange IntegroPectin reveal that these new orange pectins are comprised of pectin rich in RG-I hairy regions functionalized with citrus flavonoids and terpenes. IntegroPectin from bitter orange is high methoxyl, whereas blood IntegroPectin is low methoxyl pectin. Following the first investigation of bioproducts obtainable from HC-based extraction of *Citrus sinensis* industrial biowaste [4], the new citrus pectins obtained from bitter orange and red orange contain high amounts of hesperidin and naringin, with a uniquely high amount of flavonoids in *C. aurantium* IntegroPectin. Limonene is the dominant volatile compound found in *C. sinensis* red orange IntegroPectin. On the other hand, bitter orange IntegroPectin contains a high amount of α-terpineol. These new orange pectins, sustainably sourced using only water and electricity, can be used to produce biocompatible and antimicrobial films similar to what happens with grapefruit and lemon IntegroPectin, having broad-scope antimicrobial properties [38]. Given the wide-ranging and powerful biological activities of pectin [39], citrus flavonoids, and terpenes [40], the application potential of these new orange pectins might range from neuro- and cardiovascular protection to oral hygiene. Forthcoming studies will unveil the first biological properties of these new pectins derived by the most widely harvested citrus fruit.

## Figures and Tables

**Table 1:** Conditions employed for the SPME-GC/MS analysis.

Sample/Matrix:	0.362 g freeze-dried, ground red orange IntegroPectin; 0.683 g freeze-dried, ground bitter orange IntegroPectin
SPME fiber:	50/30 µm DVB/CAR/PDMS
Sample equilibration:	24 h, room temperature
Extraction:	2 min for red orange IntegroPectin, headspace, room temperature; 20 s for bitter orange IntegroPectin, headspace, room temperature
Column:	ZB-WAX Phenomenex, L = 30 m × I.D. = 0.25 mm × df = 0.25 µm
Oven:	50 °C (0 min), 10 °C/min to 250 °C (5 min)
Injection T:	260 °C
Detector:	Triple quadrupole
Scan range:	Full scan, *m*/*z* 50–500
Carrier gas:	He 99.9999%, 1 mL/min constant flow

**Table 2:** Decomposition results of the 850–1800 cm^-1^ region of the DRIFT spectra.

Assignment	IP-Bitter		IP-Blood	
	Center/cm^−1^	Area	Center/cm^−1^	Area
ν(C=O)_methyl-ester_	1749	3.80	1754	1.16
ν(C=O)_ester_	1717	19.80	1731	7.09
ν(C=O)_carboxylic acid_	1641	8.45	1663	9.25
ν_as_(COO^−^)	1592	8.11	1603	8.10
ν(C-O-C)_pyranose_ + ν(C-OH) + ν(C-C)	1138	2.61	1145	2.48
	1109	1.77	1110	2.28
	1081	3.61	1076	4.02
	1054	2.49	1051	0.33
	1020	4.59	1023	4.71
	989	1.53	970	0.99
	DE	0.59	DE	0.32
	HG α to	0.71	HG α to	0.63

**Table 3:** Phenolic compounds in red orange IntegroPectin, bitter orange IntegroPectin, and bitter orange IntegroPectin re-extracted at 90 °C.

Compound Class	Red Orange IntegroPectin (mg/g)	Bitter Orange IntegroPectin (mg/g)	Bitter Orange IntegroPectin Re-Extracted at 90 °C (mg/g)
**Flavonoids**			
Rutin	0.131	-	-
Naringin	2.004	47.164	9.494
Naringenin	-	0.169	-
Hesperidin	15.087	56.811	7.961
Hesperetin	-	0.173	-
Eriocitrin	-	14.765	1.690
Diosmin	-	0.286	0.055
**Phenolic acids**			
Caffeic acid	-	0.236	0.016
Gallic acid	0.015	-	-
**Biophenols (total)**	17.24	119.60	19.22

**Table 4:** Mass of the volatile analytes and fragment ions in both orange IntegroPectin samples.

Volatile Compound (Bitter Orange IntegroPectin)	Molecular Weight	Fragment Ions (*m*/*z*)
α-pinene	136	69; 77; 79; 91
1-pentanol	88	57; 56; 70; 93
trans-linalool oxide	170	59; 81; 93; 136
2,3-butanediol	90	55; 71; 72; 75
α-linalool	154	69; 80; 93; 121
1,3-butanediol	90	55; 71; 72; 75
α-terpineol	154	81; 93; 121; 136
**Volatile compound** **(red orange IntegroPectin)**		
*d*-limonene	136	67; 79; 93; 94
1,3-bis(1,1-dimethylethyl)-benzene	190	57; 176; 147; 190

**Table 5:** Volatile compounds in bitter orange IntegroPectin.

Compound	Area (%)
α-pinene	1.45
1-pentanol	1.33
trans-linalool oxide	1.07
2,3-butanediol	5.16
α-linalool	1.17
1,3 butanediol	7.26
α-terpineol	82.56
**Compound in trace**	
4-hydroxy-2-butanone	-
*d*-limonene	-
1-hexanol	-
3-hexen-1-ol	-
terpinen-4-ol	-
phenylethyl alcohol	-
*p*-menthane-1,8-diol	-

**Table 6:** Volatile compounds in red orange IntegroPectin.

Compound	Area (%)
*d*-limonene	90.71
1,3-bis(1,1-dimethylethyl)-benzene	2.49
2,3-butaniedol	0.32
α-linalool	1.05
1,3 butaniedol	5.43
**Compound in trace**	
2,6-dimethyl-nonanol	-
terpinen-4-ol	-
phenylethyl alcohol	-

## Data Availability

All spectra are available by contacting the corresponding authors.

## Supplementary Materials

The following supporting information can be downloaded at: https://www.mdpi.com/article/10.3390/molecules27103243/s1, Figure S1: Bitter orange IntegroPectin (left) and red orange IntegroPectin (right) after the extraction with aqueous EtOH at room temperature; Figure S2. Chromatogram of bitter orange IntegroPectin extract. Figure S3. Bitter orange IntegroPectin extract: mass spectra of naringenin (a), naringin (b), and eriocitrin (c). Figure S4. Bitter orange IntegroPectin extract: mass spectra of hesperidin (a), and caffeic acid (b). Figure S5. Chromatogram of red orange IntegroPectin. Figure S6. Red orange IntegroPectin extract: mass spectra of naringin (a), gallic acid (b), and hesperidin (c).
