# Supplementary material for: Red Orange and Bitter Orange IntegroPectin: Structure and Main Functional Compounds
Source: Molecules. 2022 May 19;27(10):3243. doi: 10.3390/molecules27103243 (PMC9147265; doi:10.3390/molecules27103243)
Supplement: Supplementary file 1 [file molecules-27-03243-s001.zip › molecules-1682048-supplementary.pdf]

Article

Red Orange and Bitter Orange IntegroPectin: Structure and Main Functional Compounds

Antonino Scurria [1,2], Marzia Sciortino [3], Ana Rosa Garcia [4], Mario Pagliaro [1,*], Giuseppe Avellone [3], Alexandra Fidalgo [4], Lorenzo Albanese [5], Francesco Meneguzzo [5], Rosaria Ciriminna [1,*] and Laura M. Ilharco [4,*]

[1] Istituto per lo Studio dei Materiali Nanostrutturati, CNR, Via U. La Malfa 153, 90146 Palermo, Italy; antonino.scurria@ismn.cnr.it
[2] Dipartimento DICEAM, Università degli Studi "Mediterranea" di Reggio Calabria, Via Graziella, Loc. Feo di Vito, 89122 Reggio Calabria, Italy
[3] Dipartimento di Scienze e Tecnologie Biologiche Chimiche e Farmaceutiche, Università di Palermo, Via Archirafi 32, 90123 Palermo, Italy; marziasciortino@gmail.com (M.S.); beppe.avellone@unipa.it (G.A.)
[4] Institute of Bioscience and Biotechnology, Instituto Superior Técnico, Universidade de Lisboa, Avenida Rovisco Pais 1, 1049-001 Lisboa, Portugal; argarcia@tecnico.ulisboa.pt (A.R.G.); alexandra.m.abrantes.fidalgo@gmail.com (A.F.)
[5] Istituto per la Bioeconomia, CNR, Via Madonna del Piano 10, 50019 Sesto Fiorentino, Italy; lorenzo.albanese@cnr.it (L.A.); francesco.meneguzzo@cnr.it (F.M.)
* Correspondence: mario.pagliaro@cnr.it (M.P.); rosaria.ciriminna@cnr.it (R.C.); lilharco@tecnico.ulisboa.pt (L.M.I.)

Supplementary Information

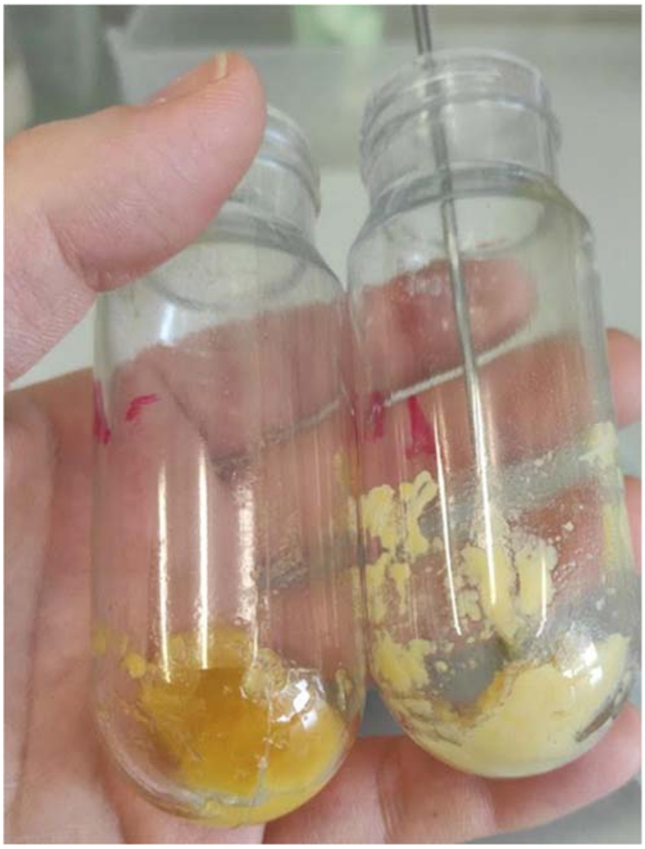

Figure S1. Bitter orange IntegroPectin (*left*) and red orange IntegroPectin (*right*) after the extraction with aqueous EtOH at room temperature.

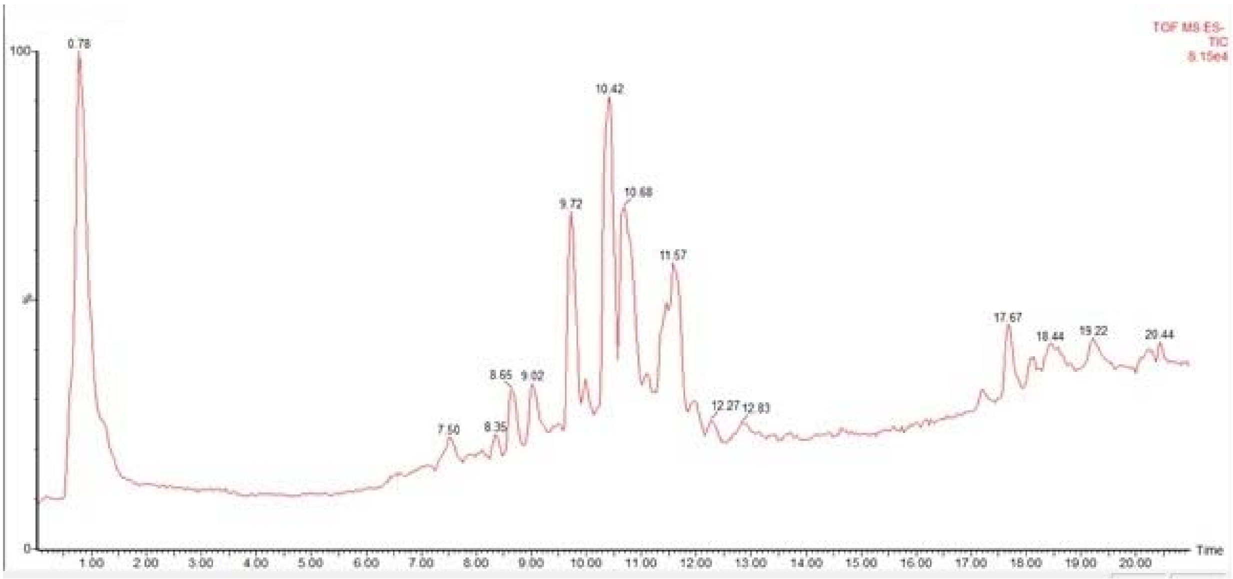

Figure S2. Chromatogram of bitter orange IntegroPectin extract.

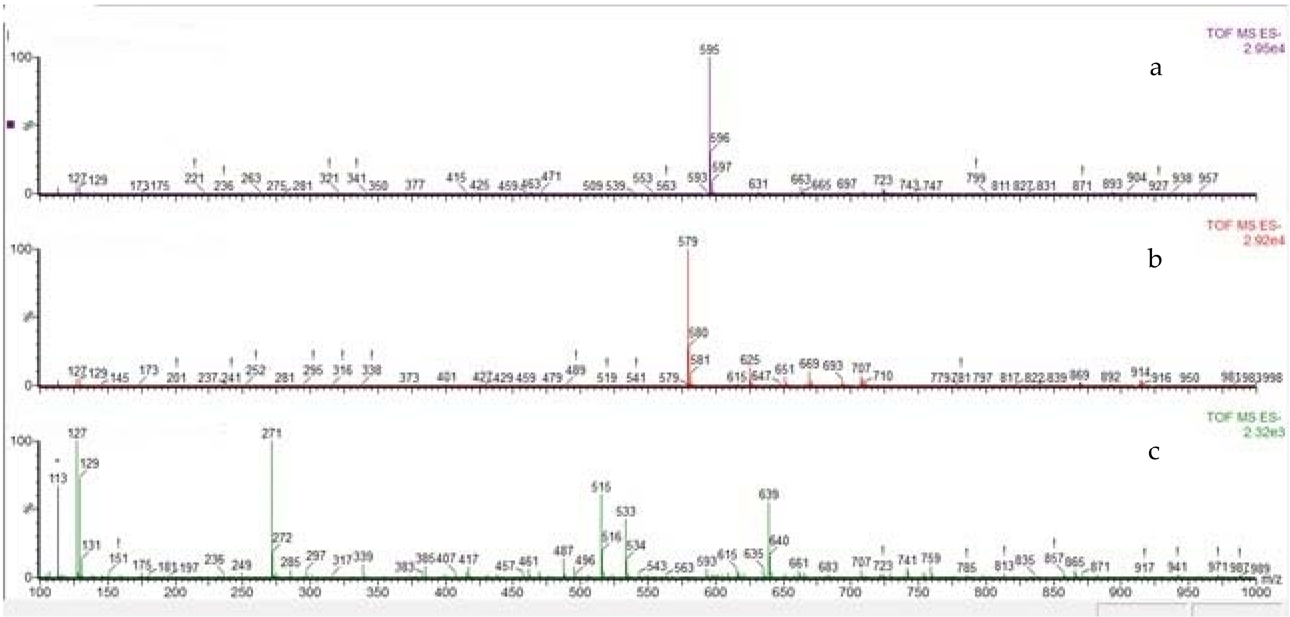

Figure S3. Bitter orange IntegroPectin extract: mass spectra of naringenin (a), naringin (b), and eriocitrin (c).

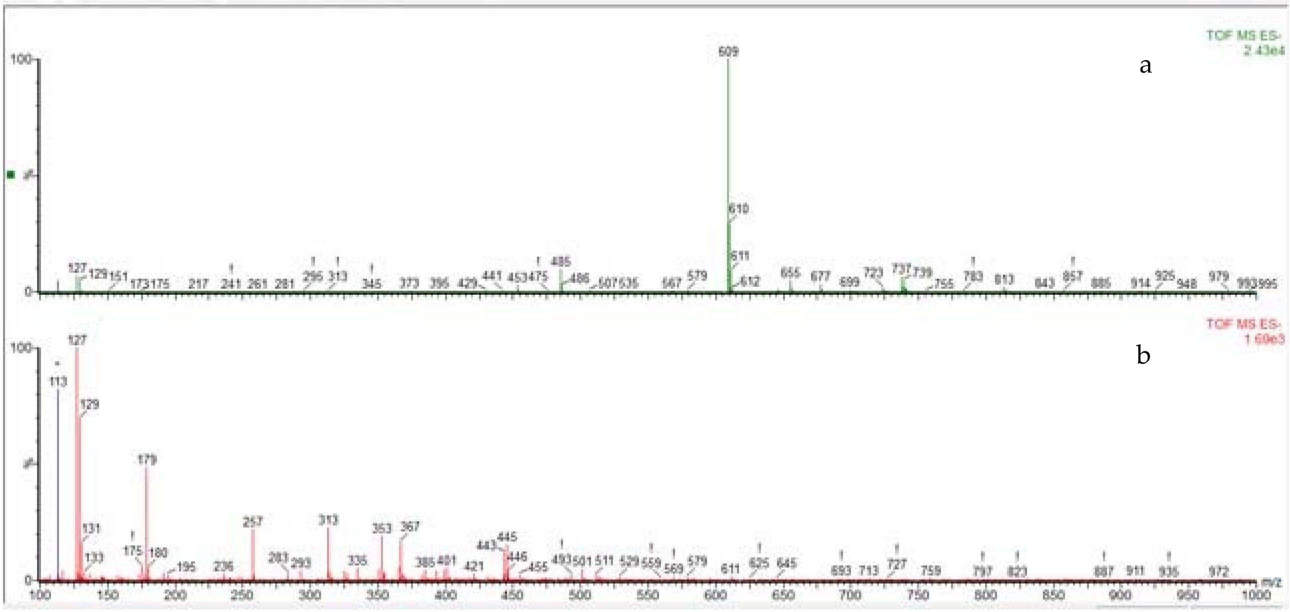

Figure S4. Bitter orange IntegroPectin extract: mass spectra of hesperidin (a), and caffeic acid (b).

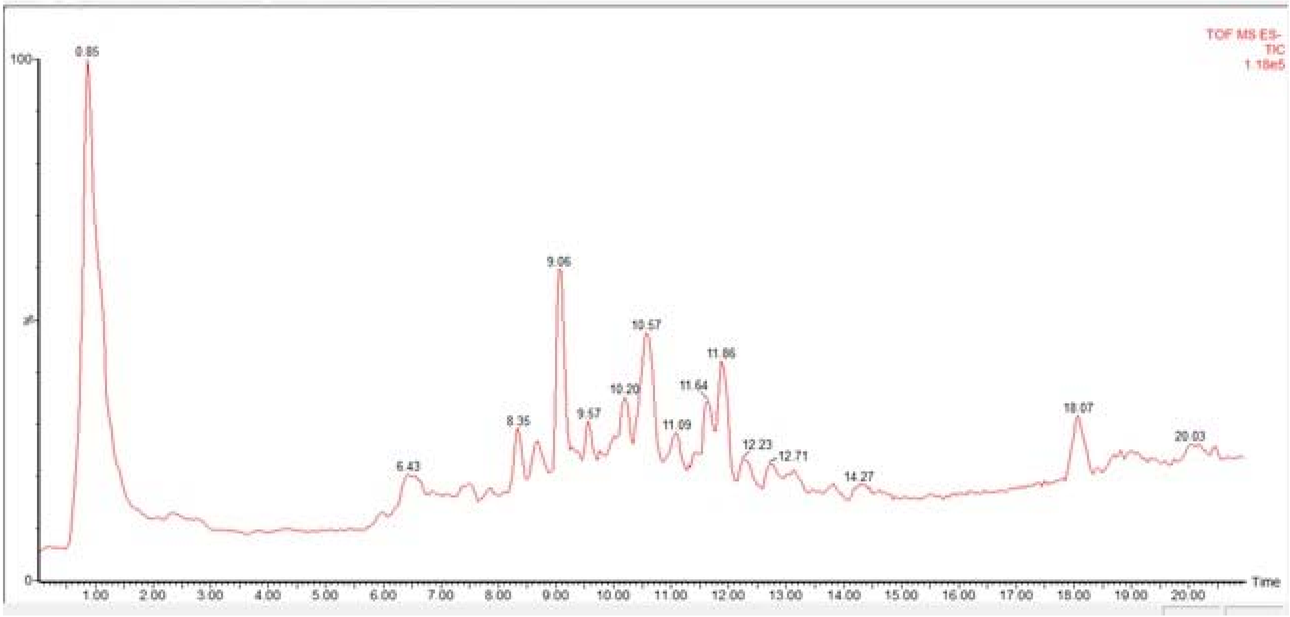

Figure S5. Chromatogram of red orange IntegroPectin

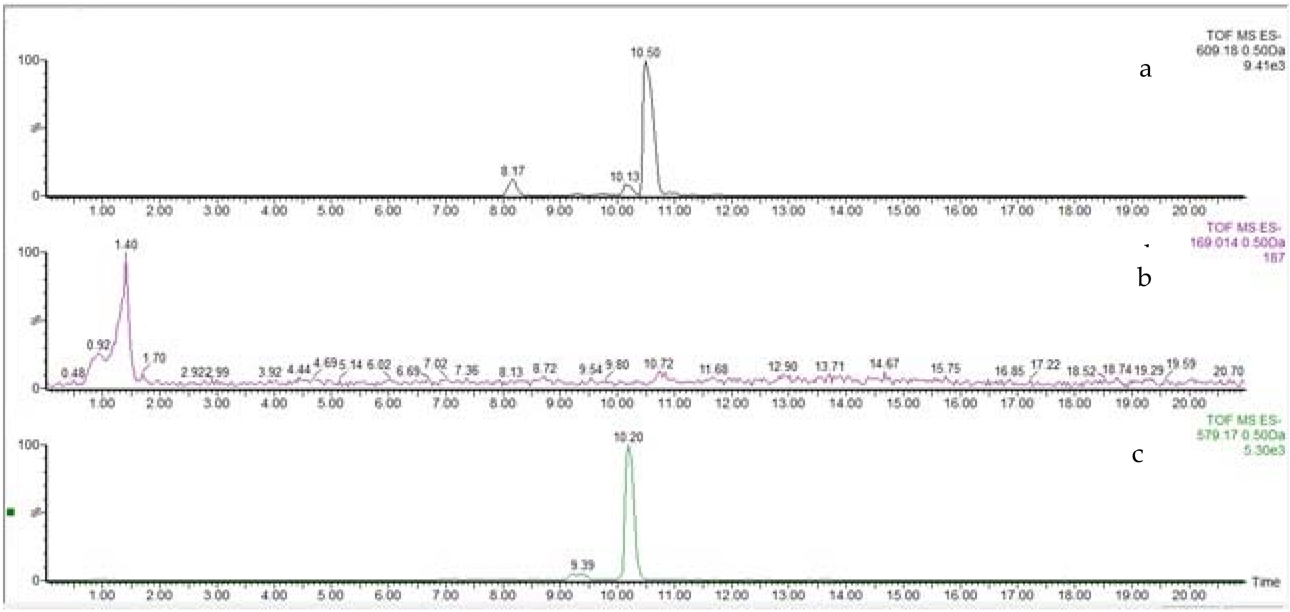

Figure S6. Red orange IntegroPectin extract: mass spectra of naringin (a), gallic acid (b), and hesperidin (c).
